# How the adaptation of the human microbiome to harsh space environment can determine the chances of success for a space mission to Mars and beyond

**DOI:** 10.3389/fmicb.2023.1237564

**Published:** 2024-02-08

**Authors:** Seyed Mohammad Javad Mortazavi, Ilham Said-Salman, Ali Reza Mortazavi, Sami El Khatib, Lembit Sihver

**Affiliations:** ^1^Ionizing and non-ionizing radiation protection research center (INIRPRC), Shiraz University of Medical Sciences, Shiraz, Iran; ^2^Department of Biological and Chemical Sciences, School of Arts & Sciences, Lebanese International University, Saida, Lebanon; ^3^Department of Biological and Chemical Sciences, International University of Beirut, Beirut, Lebanon; ^4^MVLS College, The University of Glasgow, Glasgow, United Kingdom; ^5^Department of Biomedical Sciences, School of Arts and Sciences, Lebanese International University, Beirut, Lebanon; ^6^Center for Applied Mathematics and Bioinformatics (CAMB) at Gulf University for Science and Technology, Kuwait City, Kuwait; ^7^Department of Radiation Dosimetry, Nuclear Physics Institute (NPI) of the Czech Academy of Sciences (CAS), Prague, Czechia; ^8^Department of Radiation Physics, Technische Universität Wien Atominstitut, Vienna, Austria

**Keywords:** human microbiome, space environment, space radiation, resistance to antibiotics, microorganism

## Abstract

The ability of human cells to adapt to space radiation is essential for the well-being of astronauts during long-distance space expeditions, such as voyages to Mars or other deep space destinations. However, the adaptation of the microbiomes should not be overlooked. Microorganisms inside an astronaut’s body, or inside the space station or other spacecraft, will also be exposed to radiation, which may induce resistance to antibiotics, UV, heat, desiccation, and other life-threatening factors. Therefore, it is essential to consider the potential effects of radiation not only on humans but also on their microbiomes to develop effective risk reduction strategies for space missions. Studying the human microbiome in space missions can have several potential benefits, including but not limited to a better understanding of the major effects space travel has on human health, developing new technologies for monitoring health and developing new radiation therapies and treatments. While radioadaptive response in astronauts’ cells can lead to resistance against high levels of space radiation, radioadaptive response in their microbiome can lead to resistance against UV, heat, desiccation, antibiotics, and radiation. As astronauts and their microbiomes compete to adapt to the space environment. The microorganisms may emerge as the winners, leading to life-threatening situations due to lethal infections. Therefore, understanding the magnitude of the adaptation of microorganisms before launching a space mission is crucial to be able to develop effective strategies to mitigate the risks associated with radiation exposure. Ensuring the safety and well-being of astronauts during long-duration space missions and minimizing the risks linked with radiation exposure can be achieved by adopting this approach.

## 1 Introduction

In this article, the impact of radiation on the human microbiome during space expeditions is discussed. The authors note that the microbiota of astronauts will encounter numerous protons prior to HZE particles, which could potentially enhance their resilience to radiation. However, bacterial exposure to low-level radiation may also induce resistance to antibiotics, which could be life-threatening for the astronauts. It is suggested that estimating the magnitude of adaptation in microorganisms before launching a space mission could be crucial for developing risk reduction strategies.

The article also discusses the potential competition between humans and their microbiomes for adaptation to the space environment. Bacteria may enter into a dormant state, so these microorganisms can maintain some metabolic activity but are unable to divide because of their limited ability to adapt (Rittershaus et al., 2013). Bacterial gene expression may be modified to resist stress conditions by shutting down metabolism and arresting growth to survive. In reaction to environmental stress, various methods of persistence can be initiated, including the SOS response, DNA repair, pump efflux, and biofilm formation (Dörr et al., 2009; Wood et al., 2013; Cui et al., 2018; Said-Salman et al., 2019).

Overall, the article highlights the potential risks associated with radiation exposure in space missions and the need to understand the magnitude of adaptation in microorganisms to develop effective risk reduction strategies. It also emphasizes the importance of considering the potential effects of radiation not only on humans but also on their microbiome. Further studies are needed to investigate the mechanisms of adaptation in microorganisms and to develop strategies to mitigate the risk of radiation exposure during space missions.

## 2 Radioadaptation of humans in space

During manned space missions, microorganisms accompany humans (Mortazavi et al., 2020a). According to a NASA report from 2016, astronauts’ cells will encounter multiple proton hits before being exposed to high charge and energy (HZE) particles (Huff et al., 2016). Sequential exposure to these particles may significantly increase resistance to radiation. Research indicates that exposure to low-dose ionizing radiation can lead to the development of an adaptive cellular response, providing protection against subsequent exposure (Bhattacharjee and Ito, 2001; Mortazavi et al., 2003b; Elmore et al., 2011; Rithidech et al., 2012). This finding is significant for understanding the risks associated with space travel.

In their 2018 commentary, Bevelacqua and Mortazavi (2018) discussed the proposed ground-based radioadaptation tests. Previous findings reveal that individuals have varying levels of radioadaptation, with some showing no adaptation and even experiencing adverse biological effects prior to embarking on a deep space mission, astronaut candidates could undergo screening and testing to assess their level of radioadaptation. Those with a significant level of radioadaptation would be well-suited for such a mission. While on the mission, astronauts would encounter chronic GCR exposure, which could potentially enhance their radiation tolerance. This would prove beneficial in the event of sudden high doses of energetic particles from SPE, which could penetrate even modest spacecraft shielding (Bevelacqua and Mortazavi, 2018).

Welsh et al. (2019) have published an article in the Journal of Biomedical Physics and Engineering discussing how telomere length in space can serve as a biomarker for adaptive response. The article explores the conflicting findings of NASA and individuals residing in high background radiation areas. While exposure to high levels of ionizing radiation in India did not affect telomere length, limited data from NASA astronaut Scott Kelly suggests that exposure to space radiation can cause telomeres to lengthen, only to shorten again upon returning to Earth. This difference in outcomes may be attributed to variations in radiation dose, dose-rate, and/or radiation type. Additionally, cumulative radiation could be significantly increased during spacewalks (EVA) (Welsh et al., 2019).

**Figure 1:**
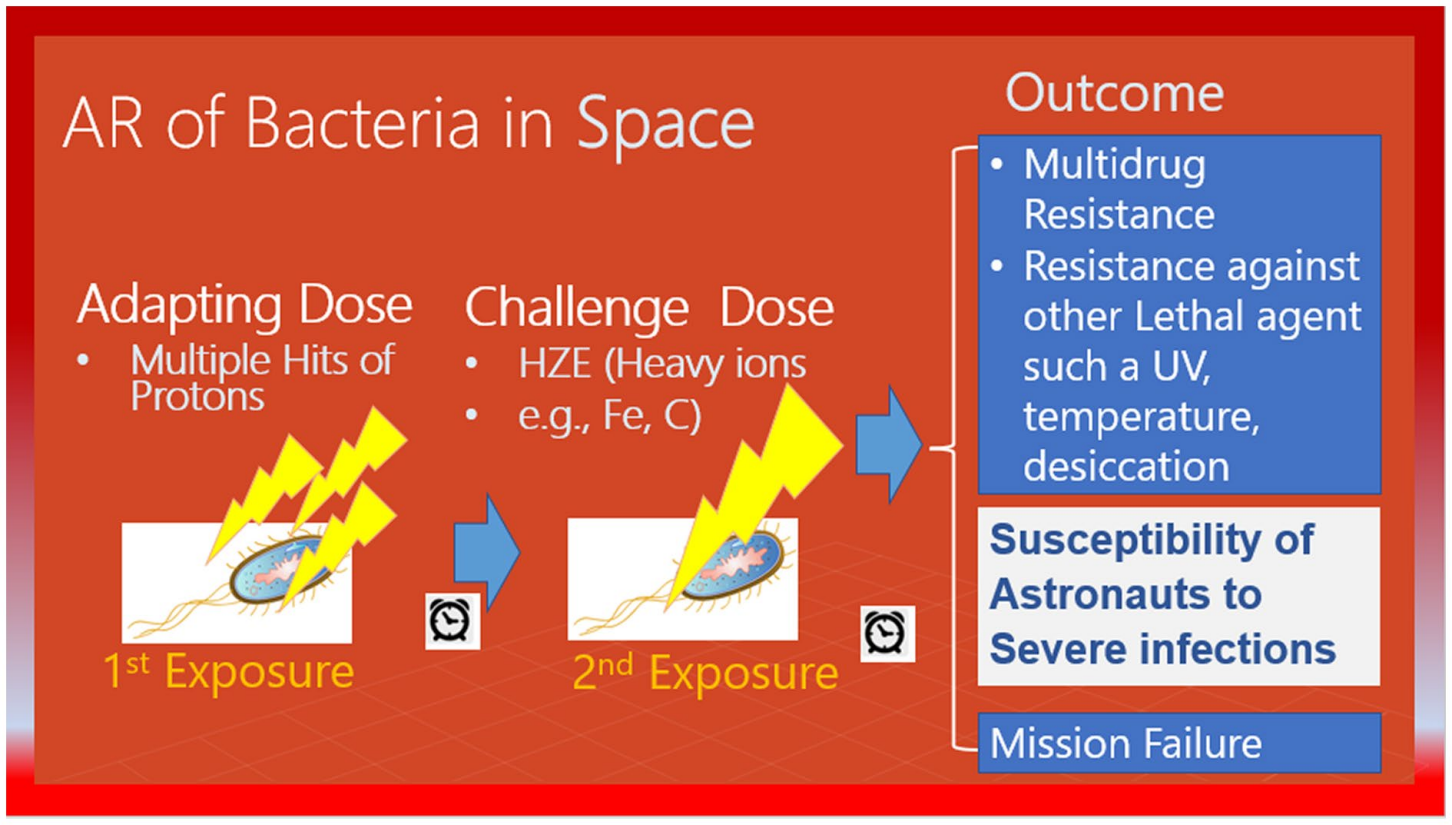
A possible scenario for the adaptive response of bacteria in space. Pre-exposure of bacteria to a low-level stressor, such as multiple hits of protons, may increase their resistance against a subsequent high-level stressor, such as heavy ions.

**Figure 2:**
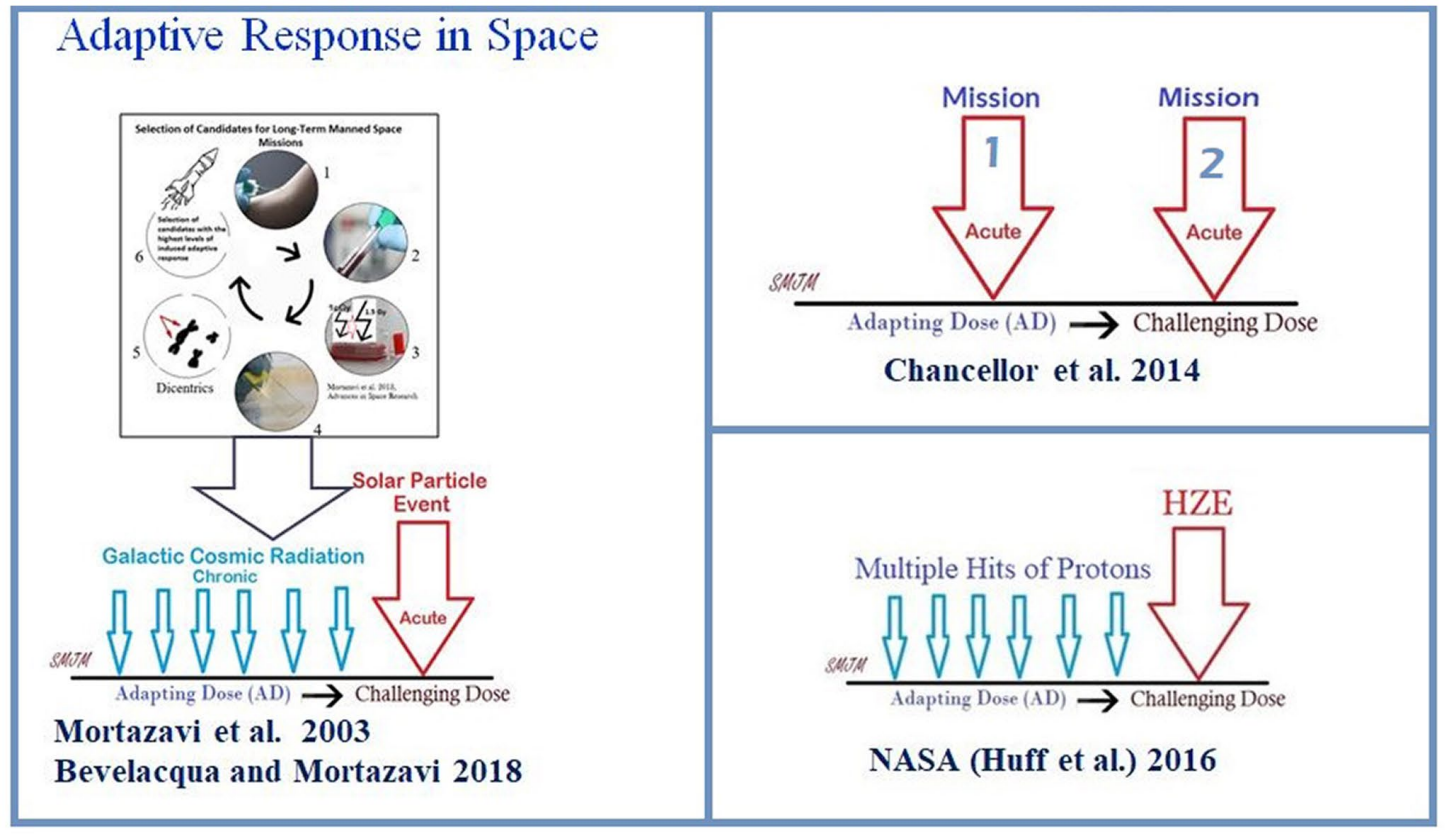
Three different potential models of adaptive response in a real space environment.

In the articles published thus far, it is important to note that three different potential types of adaptive response in a real space environment (Figure 1) have been introduced as follows:

1. In 2003, the adaptive response was introduced as an effective way to reduce radiation risk during long-term manned space missions (Mortazavi et al., 2003a). However, in 2018, Bevelacqua and Mortazavi (2018) proposed a more practical approach to this model. They suggested that before any long-term space mission, potential crew members should undergo routine cytogenetic tests to measure their adaptive response. Only those with a high adaptive response should be selected after exposure of their blood lymphocytes to an adapting low dose and later to a challenging high dose. This would help protect astronauts against chronic exposure to elevated levels of space radiation and sudden increases in flux due to solar particle events (Figure 2).
2. According to the NASA model of adaptive response in space, it is crucial to comprehend the risks associated with the space environment as it mostly consists of protons (Huff et al., 2016). The consequence is significant because cells may encounter several instances of proton exposure before being crossed by an HZE particle, which could have implications for adaptive response (Huff et al., 2016).
3. The Multiple Missions Model suggests that the first mission can be an adapting dose. The authors suggest that a second spaceflight does not increase the chances of genetic abnormalities as much as expected, which could mean that the body may have a natural defense mechanism against radiation exposure, also called a “radio-adaptive response” (Chancellor et al., 2014).

## 3 Radioadaptation of microorganisms in space

The International Space Station (ISS) is a unique and controlled system to study the interplay between the human microbiome and the microbiome of their habitats. The ISS is a hermetically sealed closed system, yet it harbors many microorganisms. The environment of galactic cosmic rays (GCR) is mainly made up of protons, so it is probable that microorganisms will be struck by multiple protons before being crossed by an HZE particle. In a study, researchers firstly exposed confluent normal human fibroblast cultures to a priming radiation dose of energetic low-LET protons (Buonanno et al., 2015). Then, after a time interval of 0, 6, or 24 h, they were subjected to a challenge dose of high-LET iron ions. The study revealed that the protective effect of the priming dose continued for a minimum of 24 h, indicating the interaction between biological effects caused by direct cellular traversal by radiation and bystander effects in cell populations exposed to mixed radiation fields. The research demonstrated that protective adaptive responses can extend from cells that are targeted by low-LET space radiation to bystander cells located nearby (Buonanno et al., 2015). However, the ability of a human cell or a microorganism to survive a proton or HZE hit can vary significantly, and the chances of survival are linked to the process of adaptation. While the effects of pre-exposure to HZE particles and/or protons in human cells are studied, there is no significant data on radioadaptation in bacteria. In this context, NASA scientists did not consider that adaptation is not limited to astronauts and radiation exposure to bacteria inside an astronaut’s body or that bacteria inside the space station could induce resistance not only to high levels of DNA damage caused by HZEs but also to other bacterial activity-threatening factors such as antibiotics (Mortazavi et al., 2020b), as shown in Figure 2.

## 4 Effects of the spaceflight environment on the microbiome

The main stressors in space include radiation, microgravity, isolation, confinement, and distance from the Earth. However, regarding human microbiome, space radiation and microgravity are the key stressors. A microbiome is usually defined by the entire habitat, including the microorganisms and the surrounding environmental conditions (Marchesi and Ravel, 2015).

### 4.1 Single stressor ground studies

**Figure 3:**
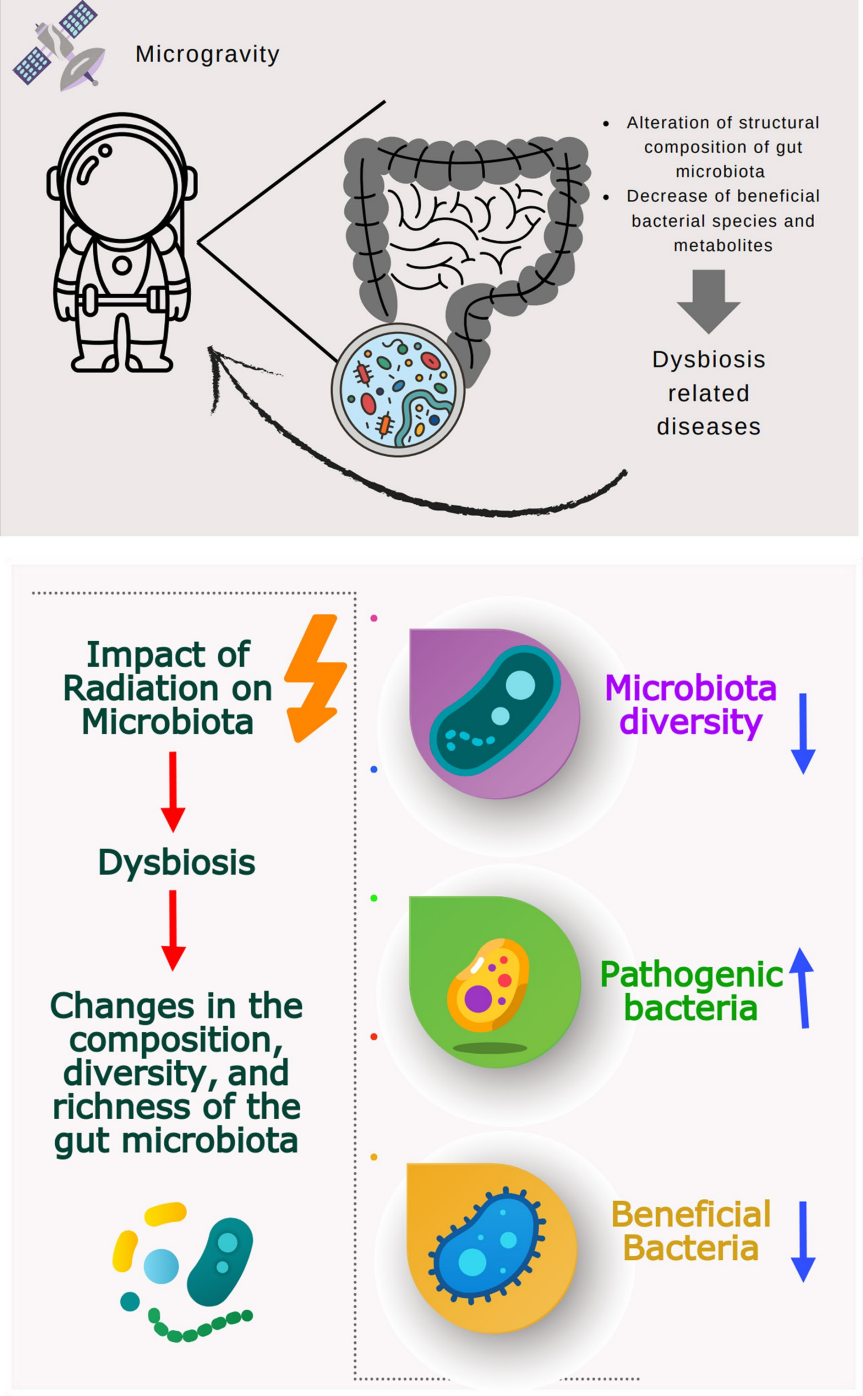
Potential effect of simulated microgravity on astronaut’s microbiome.

Many studies investigating the effects of simulated microgravity on different microorganisms (bacteria and fungi) are summarized in Table 1. Microgravity may affect various cellular functions, such as cell morphology, growth rate, biofilm formation, gene expression, microbial mutations, antibiotic resistance, virulence, and resistance to adapt to simulated microgravity (Table 1). Moreover, microgravity stress has been reported to alter bacterial microbiome diversity and composition in animal models (Wang et al., 2020; Siddiqui et al., 2022; Yuan et al., 2023). Disruption of microbiome and metabolites may be associated with dysbiosis-related diseases such as inflammatory bowel disease, obesity, autism, allergy, diabetes, and some gastrointestinal cancers (Degruttola et al., 2016; Figure 3).

**Table 1:** Bio-effects of microgravity on various cellular functions of bacteria.

Bacterial strains	Bio-effect	Reference
*Escherichia coli*	Enhanced bacterial proliferation	Kacena et al. (1999a)
*Escherichia coli and Bacillus subtilis*	Shorter lag-phase, increased log, and exponential phases.	Kacena et al. (1999b)
*Streptomyces hygroscopicus*	Lowered dry cell weight and inhibition of rapamycin production.	Fang et al. (2000)
*Pseudomonas aeruginosa*	No differences in the growth curves or membrane polarization values.	England et al. (2003)
*Escherichia coli*	Increased biofilm formation and resistance to the general stressors salt and ethanol and to two antibiotics (σ^s^ dependent)	Lynch et al. (2006)
*Escherichia coli*	Conferred resistance by Affecting σ^s^ synthesis and increasing *rpoS* mRNA translational efficiency.	Lynch et al. (2004)
*Chryseobacterium* sp.*, Pseudomonas fluorescens and Stenotrophomonas maltophilia*	Differences in motility among isolates, higher growth of planktonic cells but no change in biofilm formation.	Baker and Leff (2005a)
*Stenotrophomonas paucimobilis and Acinetobacter radioresistens*	No differences in the growth of *Acinetobacter radioresistens* whereas *S. paucimobilis* was negatively affected by modeled reduced gravity.	Baker and Leff (2005b)
*Saccharomyces cerevisiae*	Altered gene expression associated with the establishment of polarity and cell separation	Purevdorj-Gage et al. (2006)
*Streptococcus pneumoniae*	101 differentially expressed genes implicated in the adaptation to low-shear modeled microgravity	Allen et al. (2007)
*Salmonella typhimurium*	167 differentially expressed genes and 73 proteins changed expression with Hfq identified as a regulator involved in the adaptation to microgravity	Wilson et al. (2007)
*Saccharomyces cerevisiae*	Affected cell growth and gene expression.	Coleman et al. (2007)
*Staphylococcus aureus*	Significant changes in global gene expression, no change in antibiotic susceptibly and reduction in the expression of virulence elements.	Rosado et al. (2010)
*Escherichia coli, Staphylococcus epidermis*	Increased growth rate and oxygen availability.	Dijkstra et al. (2011)
*Saccharomyces cerevisiae*	Reduced invasive growth in the center of the yeast colony and bud scar distribution was slightly affected.	Van Mulders et al. (2011)
*Saccharomyces cerevisiae*	Microgravity is “sensed” by yeast cells as a stress condition and several signaling pathways are activated.	Willaert (2013)
*Escherichia coli*	Enhanced growth rate and alteration of gene expression of hundred genes	Arunasri et al. (2013)
*Pseudomonas aeruginosa*	Higher cell densities under nutrient-limiting conditions and microgravity conditions	Kim et al. (2013)
*Candida albicans*	Enhanced cell aggregation and random budding	Crabbé et al. (2013)
*Escherichia coli*	5 induced mutations in coding sequences.	Tirumalai et al. (2017)
*Enterobacter bugandensis and Staphylococcus haemolyticus isolated from ISS*	Resistance of *Enterobacter bugandensis* to all 9 antibiotics tested whereas no antibiotic resistance was observed in *Staphylococcus haemolyticus*	Urbaniak et al. (2018)
*Escherichia coli*	Upregulated of differentially stress response genes including oxidative stress genes.	Aunins et al. (2018)
*Escherichia coli*	Acquired antibiotic resistance	Tirumalai et al. (2019)
*Listeria monocytogenes*	Alterations in *L. monocytogenes* stress and virulence response, due to changes in lipid composition and related gene expression.	Sheet et al. (2020)
*Escherichia coli*	Increased growth and antibiotic resistance	Allen et al. (2022)

### 4.2 Flight studies

The importance of the gut microbiota (GM) in animal models and its impact on their characteristics is becoming more obvious. Any alterations or disorders to the GM can greatly influence the phenotypes expressed by these murine models, which adds a crucial and intricate element to both basic research and pre-clinical studies. Franklin and Ericsson (2017) have examined the primary sources of variability in GM among rodent models and the versatile means to address this variability in order to improve reproducibility. The gut microbiome plays a crucial role in maintaining the overall health of the host, including astronauts during space travel. It affects the general immune system, metabolism, neurological strength, as well as the muscles, bones, and the ocular/retinal immune system. Therefore, it is essential to regulate the gut microbiome for the success of long-term space missions. One way to achieve this is potentially mediated by optimizing astronauts’ diets to include adequate amounts of dietary fiber, prebiotics, bioactive compounds, and probiotics. Alternatively, the transplantion of the fecal microbiota of the crew members into probiotic capsules before the onset of the spatial journey, allows astronauts to diversify their microbiome with their own set of microorganisms (Siddiqui et al., 2021b).

As discussed by Tronni et al., from the early 1960s and onwards, preclinical research conducted on animals and humans has shown that space stressors such as radiation, microgravity, pathogens, dietary constraints, overloads during launch and landing, constant noise, hypodynamia and hypomagnetic fields can lead to a significant imbalance in the gut microbiota. This mispropotion could then contribute to gastrointestinal diseases, metabolic imbalances, and prominent changes in bacterial physiology. This has been confirmed in both space and ground-based studies (Nickerson et al., 2004; Wilson et al., 2007; Barrila et al., 2010; Castro et al., 2011; Crabbé et al., 2011; Turroni et al., 2020). Understanding the complex interactions between the host and the gut microbiome during spaceflight is of crucial importance as it may lead to consequential interventions that help microbial communities adapt to the spaceflight-associated metabolic environment without causing harm to the mammalian physiology. Such interventions can help mitigate eminent risks to the crew’s health and performance during future long-term spaceflight missions.

In a rodent-based study, Jiang et al. (2019) reported that the gut microbiome is significantly impacted by spaceflight, likely due to microgravity and other space environmental factors. This leads to significant changes in the metabolic potential of the gut microbiota, which in turn affects the expression of metabolic genes in the host liver. The authors hypothesized that microgravity alters the metabolic environment of the gut microbes by affecting fluid shear dynamics and digestive peristaltinc movement, as well as the physiological and behavioral responses of the host. Jiang et al. (2019) believes that the gut microbiota adapts to these changes by altering community structure and genetic content, which can have a substantial impact on the host’s biological functions. Consequently, changes in the gut microbiota are a crucial aspect of mammalian adaptation to the space environment.

Using a ground-based microgravity hindlimb unloading (HU) mouse model, Siddiqui et al. (2022) have shown that the amount of several bacterial species is significantly lower in mice in microgravity conditions compared to in mice on Earth. Additionally, several changes were observed perceived in the ratio of Bacteroidetes to Firmicutes under simulated microgravity conditions. Accordingly, the authors suggested that in future studies, it would be crucial to isolate the bacterial species that showed a significant decrease in number and investigate their metabolites in order to develop pre/probiotics. Siddiqui et al. have also suggested that since it is difficult to directly test the GM in astronauts, the species that were already identified in the “astronaut microbiome project,” such as Akkermansia, could also be tested *in vivo in vivo* mice as pre/probiotics. Other studies have suggested a strong association between dysbiosis and variations in the glucose metabolism in the HU model, underlining the significance of assessing the gut microbiota in astronauts and its impact on glucose metabolism (Wang et al., 2019).

## 5 Why studying the human microbiome in space is of crucial importance

Studying the human microbiome in space missions can have several potential benefits, including:

### 5.1 The impact of space travel on human health

One important aspect to consider is the impact of space travel on human health, particularly the role of the microbiome in maintaining overall well-being (Cho and Blaser, 2012). Space flight can disrupt the balance of microorganisms in the body (Siddiqui et al., 2021a). Studying the microbiome in space can help scientists better understand how space travel affects human health and develop strategies to mitigate any negative effects.

### 5.2 Developing new technologies for monitoring health

Studying the microbiome during space missions has the potential to contribute to the advancement of innovative technologies for tracking and maintaining the health of astronauts. For example, researchers are exploring the use of “smart toilets” to analyze crew microbiomes and detect health problems early on Ge et al. (2022, 2023) and Tasoglu (2022).

### 5.3 Developing new therapies and treatments

Exploring the microbiome in space has the potential to pave the way for novel therapies and remedies for various health ailments. For example, researchers are exploring the use of probiotics and other microbiome-based therapies to prevent or treat conditions such as bone loss and immune system dysfunction that can occur during space travel (Turroni et al., 2020; Bharindwal et al., 2023).

### 5.4 Advancing our understanding of microbiomes in general

Studying the microbiome in space can also help advance our understanding of microbiomes in general, including how they interact with the environment and how they evolve over time. This knowledge can have far-reaching implications on Earth for fields such as medicine and agriculture.

## 6 Potential benefits of studying astronaut’s microbiome

The astronaut’s microbiome refers to the collection of microorganisms, such as bacteria, viruses, and fungi, residing in an astronaut’s body. The microbiome probably undergoes alterations in both composition and functionality while in space (Turroni et al., 2020). During space travel, besides the key issue of altered diet, astronauts are exposed to unique stressors, such as microgravity, radiation, and changes in their environment, which can potentially alter the composition of their microbiomes and have a negative impact on their health. It is worth noting that the interactions among these stressors and an astronaut’s body are complex and not fully explored yet. According to Voorhies et al. (2019), the astronaut microbiome is affected by long term spaceflight and the microbial composition of astronaut skin is comparable to that of ISS environmental surfaces. ISS atmosphere has been shown to exhibit diverse microbial communities with increased antimicrobial resistance and virulence profile (Singh et al., 2018). Microbiome changes under space flight circumstances were also confirmed by Avila-Herrera et al. (2020) thorough examination of the microbiome of crew members’ saliva and its associated ISS environmental microbiome using metagenomic sequencing. While no significant change was reported by NASA research conducted on twins tracking their microbiome changes prior to, during, and after flight (Garrett-Bakelman et al., 2019). One potential benefit of studying the astronauts’ microbiome in space is to understand how space travel affects the composition and diversity of the microbiome. Research has shown that the microbiome can undergo changes in response to factors such as diet, stress, and environmental conditions. The unique conditions of space, including microgravity, radiation exposure, and altered diet, can potentially disrupt the balance of the microbiome. By studying the changes in the microbiome during space travel, scientists can gain insights into how these alterations may impact astronaut health and develop strategies to mitigate any negative effects.

Furthermore, studying the microbiome can help researchers understand the impact of space travel on the immune system. The microorganisms that inhabit different areas of the human body play a crucial role in maintaining good health by mechanisms such as producing essential vitamins (LeBlanc et al., 2013), and aiding in the development and regulation of our immune system. Changes in the composition of the gut microbiota caused by both genetic and environmental factors can raise the likelihood of pathogen infection, encourage the proliferation of harmful pathobionts, and contribute to the emergence of inflammatory disorders. So, the microbiome plays a crucial role in training and modulating the immune system (Mazmanian et al., 2005; Hyun et al., 2015), and any disruptions to the microbiome can potentially affect immune function. Understanding how space travel affects the microbiome-immune system interaction can aid in developing strategies to maintain astronaut health and prevent infections during long-duration space missions.

Another potential benefit of studying the astronauts’ microbiome in space is the potential for developing novel therapies and interventions. The unique conditions of space can lead to the growth of specific microorganisms that are not commonly found on Earth. These microorganisms may possess unique properties and capabilities that could be harnessed for various applications, including the development of new drugs, antimicrobial agents, or biotechnological advancements. By studying the microbiome in space, scientists can identify and characterize these novel microorganisms, potentially leading to the discovery of new therapeutic options. Additionally, the altered environmental conditions in space, including temperature, oxygen levels, and limitations in diffusion, offer an opportunity to optimize the production of valuable metabolites by engineered microorganisms (Javad Mortazavi et al., 2021).

However, studying the astronauts’ microbiome in space also has several barriers and challenges. One of the primary challenges is associated with conducting experiments in the microgravity environment. Substantial evidence indicates that microgravity can affect the growth and behavior of microorganisms, making it challenging to obtain accurate and reliable data. Moreover, a potential hazard of studying the astronauts’ microbiome in space is the possible spread of pathogenic microorganisms in the closed environment of a spacecraft. The confined space and recycled air systems can facilitate the transmission of pathogens, leading to the risk of infections among the crew (Welsh et al., 2020; Javad Mortazavi et al., 2021). Understanding the dynamics of the microbiome in space can help identify potential pathogens and develop strategies to prevent their spread.

## 7 Hazards associated with disrupting the human microbiome during space travel

Disrupting the human microbiome during space travel can have several potential risks, including:

### 7.1 Compromised immune function

The microbiome plays a crucial role in maintaining the immune system, and disrupting the microbiome can compromise immune function (Lambring et al., 2019). This can make astronauts more susceptible to infections and illnesses, which can be especially dangerous in the isolated and confined environment of a spacecraft (Crucian et al., 2018).

### 7.2 Nutrient absorption

The microbiome also plays a role in the digestion, absorption of nutrients and energy regulation (Krajmalnik-Brown et al., 2012). Disrupting the microbiome can lead to malnutrition, negatively impact overall health and well-being, and may impair crew performance.

### 7.3 Psychological effects

Upsetting the microbiome can impact a person’s mental state since it is believed to have a role in regulating mood and other mental health factors. This can pose a significant challenge for crew members who are already dealing with stress and other psychological issues while being isolated and confined in a spacecraft (Clapp et al., 2017).

### 7.4 Long-term effects

Finally, disrupting the microbiome during space travel can have long-term effects on crew health. For example, studies have shown that space travel can lead to changes in bone density and muscle mass that persist even after returning to Earth. It is possible that disruptions to the microbiome could also have long-term effects on crew health that are not fully understood.

## 8 Why adaptation of human microbiome in space is a concern?

In space, microorganisms present inside an astronaut’s body or within a space station. Or any other spacecraft, are likely to encounter several protons prior to being crossed by HZEs (Huff et al., 2016). As a result of this type of adaptation, their characteristics may transform to withstand the elevated levels of DNA damage caused by HZEs, as well as various antibiotics (Mortazavi et al., 2020b). As ISS transmits and receives radio signals, radiofrequency radiation can turn microorganisms resistant to antibiotics (Urbaniak et al., 2018). Consequently, radio-adaptation can occur, in both, the astronauts, and their microbiome while they are conducting a space mission. It would therefore be crucial to estimate the magnitude of the mentioned types of adaptation before the launching of the spatial mission. In the cases where the magnitude of the adaptive response could be more pronounced in the tested micro-organisms compared to in the astronauts’ cells, then, risk reduction strategies would be required.

In a recent study titled “Adaptation to space conditions of novel bacterial species isolated from the ISS revealed by functional gene annotations and comparative genome analysis” (Szydlowski et al., 2023), the researchers investigated the adaptation of microorganisms unintentionally introduced to the extreme environment of the ISS over its 20+ years of service. The study revealed that these microorganisms have undergone adaptation to life in space. The researchers observed various features in these microorganisms that are not typically found in their Earth-bound counterparts. These features enable them to thrive and adapt to the challenging conditions of space. The findings of the study suggest a convergent adaptation among the diverse microorganisms isolated from the ISS. The presence of mechanosensitive channel proteins, increased DNA repair activity, as well as metallopeptidases and novel S-layer oxidoreductases indicate a potential complementarity among these microorganisms within the context of the ISS microbiome. This study highlights the remarkable ability of microorganisms to adapt and survive in the unique and demanding environment of space. Further research in this area is crucial for understanding the implications of microbial adaptation in space travel and for ensuring the safety and success of future deep space missions.

Mortazavi et al. (2020a) published a paper in 2020 where they noted that it would be realistic to expect co-radioadaptation of astronauts’ microbiome and their bodies during deep space journeys to Mars and beyond. In their view, the race for adaptation to space between humans and their microbiome is far more important than the adaptation of microorganisms alone to the harsh environment of space. It seems evident that in contrast with humans, microorganisms have a significant advantage and will ultimately win the race for adaptation. However, this advantage of microorganisms over humans may also give rise to unexpected life-threatening situations during deep space missions.

Overall, we believe that the dynamic interplay and co-adaptation of humans and their microbiome in space travel is a crucial area of study, and understanding the potential risks and challenges associated with this relationship is of utmost importance.

## 9 The competition between humans and their microbiome for adaptation to the space environment

In a competition between astronauts and their microbiomes to adapt to the harsh space environment, microorganisms may emerge as the winners because they can evolve and adapt more quickly than humans by rapid acquisition of microbial genes (Rosenberg, 2022). Microorganisms have a much shorter generation time, enabling them to produce many more offspring, each with unique genetic mutations that can help them survive in the space environment (Wilson et al., 2007).

This rapid evolution can lead to the development of antibiotic-resistant bacteria that are difficult to treat. Additionally, microorganisms can thrive in the confined and isolated environment of a spacecraft, where they can spread easily and cause infections that can be life-threatening (Juergensmeyer et al., 1999; Aunins et al., 2018; Urbaniak et al., 2018).

**Figure 4:**
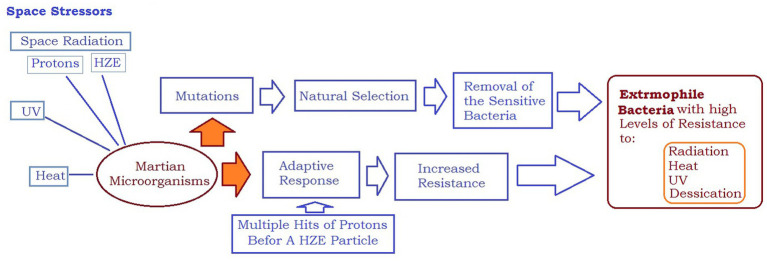
Space stressors can lead to creation of extremophile microorganisms with high levels of resistance to radiation, heat, UV, and desiccation.

**Figure 5:**
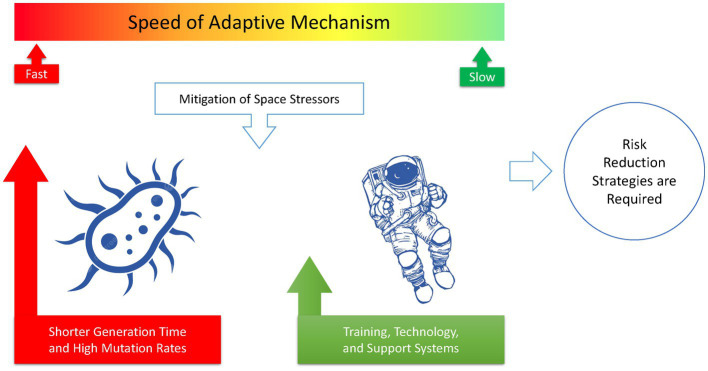
In the race for adaptation to the harsh environment of space, microorganisms have a much higher chance of success than humans. This difference underscores the need for specific risk reduction strategies to be implemented for human space exploration.

Furthermore, space travel and prolonged exposure to microgravity can weaken the human immune system, making astronauts more vulnerable to microbial infections. With the increased likelihood of antibiotic-resistant bacteria and weakened immune systems, astronauts may face significant health risks from lethal infections caused by their microbiome. Figure 4 illustrates how space stressors can induce extremophile microorganisms with high levels of resistance to radiation, heat, UV, and desiccation. The comparison shown in Figure 5 emphasizes that microorganisms are more likely to adapt successfully to the challenging conditions of space than humans. This highlights the importance of implementing risk reduction strategies that are tailored specifically for human space exploration.

To ensure the safety and health of astronauts during extended space missions, it is essential to investigate the impact of the space environment on the human microbiome and develop effective methods for managing the risks of microbial infections.

## 10 Conclusion

During deep space missions like voyages to Mars or further, it is crucial for the health of astronauts that human cells are able to adapt to radiation. However, it is equally important to consider the adaptation of the microbiome inside the astronaut’s body and inside the spacecraft. Microorganisms will also be exposed to radiation, which can induce resistance to antibiotics, UV, heat, desiccation, and other life-threatening factors. Therefore, developing effective risk reduction strategies for space missions requires studying the potential effects of radiation not only on humans but also on their microbiomes.

Studying the human microbiome in space missions can have several benefits, including understanding the effects of space travel on human health, developing new technologies for monitoring health, and developing new therapies and treatments. It is essential to note that while radioadaptive response in astronauts’ cells can lead to resistance against high levels of space radiation, radioadaptive response in astronauts’ microbiome can also lead to resistance against UV, heat, desiccation, antibiotics, and radiation. In a competition between astronauts and their microbiomes to adapt to the space environment, microorganisms may emerge as the winners, leading to life-threatening situations due to lethal infections. Therefore, understanding the magnitude of adaptation in microorganisms before launching a space mission is crucial when developing effective strategies to mitigate the risks associated with radiation exposure. By doing so, we can ensure the safety and well-being of astronauts during long-duration space missions.

## Author contributions

SMM: conceptualization and original draft preparation. IS-S and ARM: review and editing. SE: review. LS: review, editing, revision, and preparation of the submitted version. All authors contributed to the article and approved the submitted version.
